# Genetic variation corroborates subspecific delimitation in the Namib fog-basking beetle, *Onymacris unguicularis* (Haag) (Tenebrionidae, Coleoptera)

**DOI:** 10.3897/zookeys.353.6228

**Published:** 2013-11-20

**Authors:** Trip Lamb, Rachel Pollard, Jason E. Bond

**Affiliations:** 1 Department of Biology, East Carolina University, Greenville, NC 27858, USA; 2 Department of Biological Sciences and Auburn University Museum of Natural History, Auburn University, Auburn, AL 36849, USA

**Keywords:** Subspecies, integrative taxonomy, Namib Desert, *Onymacris*, Tenebrionidae

## Abstract

The fog-basking beetle, *Onymacris unguicularis* (Haag, 1875), is currently listed as a polytypic form comprising two subspecies. A flightless substrate specialist, the beetleis endemic to vegetationless dunes in the Namib, where southern populations constitute the nominate subspecies, *O. u. unguicularis*, and populations some 300 km to the north compose *O. u. schulzeae* Penrith, 1984. Their taxonomic descriptions are based on minor differences in pronotal and prosternal shape, and the phylogenetic validity of these subspecies has yet to be ascertained. Here we reassess the polytypic status of *O. unguicularis* by (1) examining diagnostic phenotypic characters in conjunction with a geometric morphometric analysis, and (2) conducting phylogenetic analysis of mitochondrial DNA sequences. Our results confirm pronotal and prosternal differences, which are complemented by geometric morphometric resolution of the subspecies. Phylogenetic analysis recovered two reciprocally monophyletic lineages that exhibit perfect phylogeographic congruence with phenotypic variation. Our genetic data identify southern and northern populations as distinct lineages, corroborate morphometric data regarding subspecific delimitation, and therefore support the recognition of *O. u. unguicularis* and *O. u. schulzeae* as valid taxa under the general lineage concept.

## Introduction

Darkling beetles (family Tenebrionidae) figure prominently in the arthropod fauna of Africa’s Namib Desert, where they compose ~80% of all coleopterans (Louw 1983). Many exhibit unique adaptations to the Namib’s substrate, thermal, and moisture conditions (Endrödy-Younga 1978; Seely et al. 2005), the most remarkable of which involves water-gathering behavior practiced by the fog-basking beetle, *Onymacris unguicularis* (Hamilton and Seely 1976). As its common name implies, *Onymacris unguicularis* ‘basks’ in the advective fogs that characterize this coastal desert and provide an important water source for Namib biota in general (Henschel and Seely 2008). Fog basking typically occurs before dawn, at which time these otherwise diurnal beetles ascend the dunes (at temperatures 20–30°C below optimal activity conditions), tilt headwards into incoming fog, and drink condensate that forms on their dorsum (Hamilton and Seely 1976; Seely et al. 1983). Although fog basking has been observed in a second species from the northern Namib (*Onymacris bicolor* (Haag, 1875)), investigations have centered largely on *Onymacris unguicularis* (Seely et al. 1983, Nøgaard and Dacke 2010; Nøgaard et al. 2012), making it one of the more widely recognized beetles worldwide.

**Figure 1.**
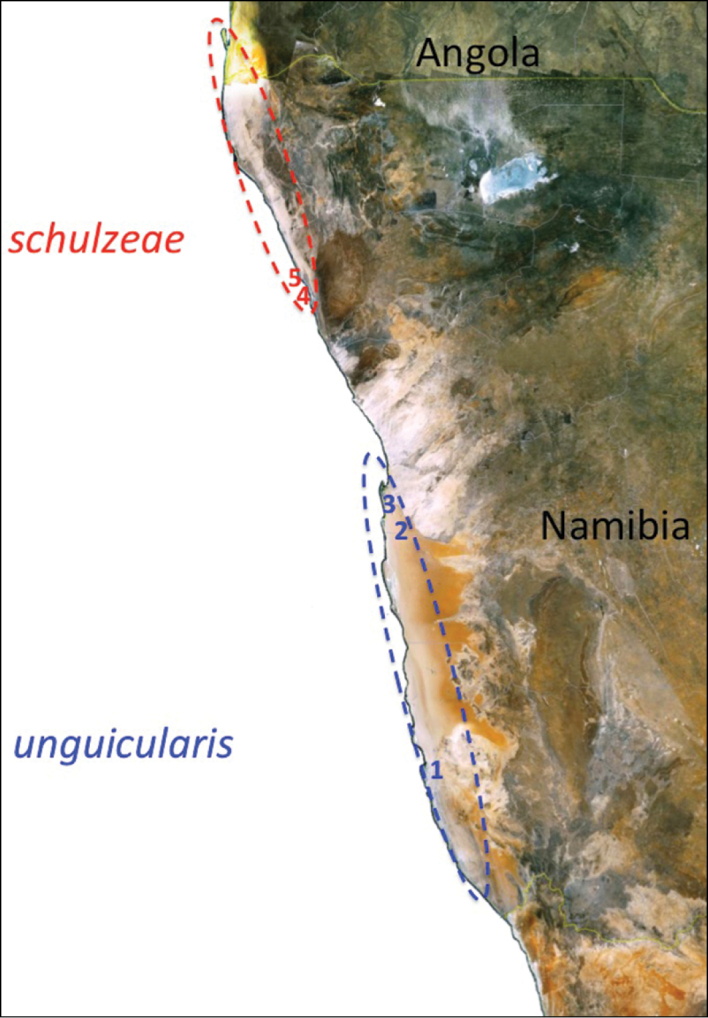
Map illustrating the range and disjunct distribution of *Onymacris unguicularis* in the Namib Desert. Subspecies distributions are approximated by oval overlays; localities for genetic sampling, listed from south to north, are: **1** Luderitz **2** Gobabeb **3** Walvis Bay, and **4, 5** Torra Bay.

**Figures 2–3.**
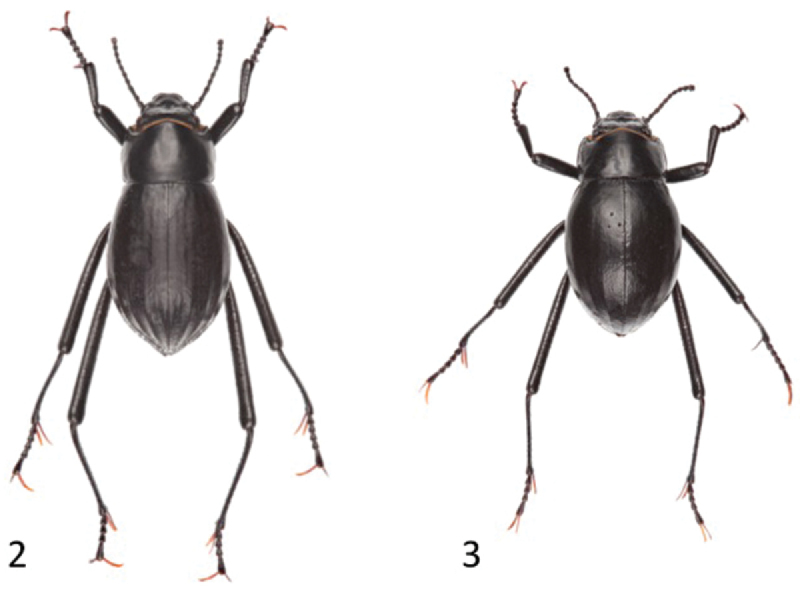
Dorsal habitus of *Onymacris unguicularis unguicularis* (**2**) and *Onymacris unguicularis schulzeae* (**3**).

*Onymacris unguicularis* has also been the subject of taxonomic investigation; Penrith (1984) examined morphological variation throughout the species’ range, which is apportioned south to north in a patchy network along the Namib’s coastal segment (Fig. 1). Ecologically, these flightless beetles exhibit further restriction, being habitually if not exclusively confined to vegetationless dunes within the desert’s major sand seas. Penrith (1984) identified phenotypic distinctions between northern vs. southern populations, which are separated by ~300 km of duneless plains. Based on their morphological differentiation and apparent absence of gene flow, she proposed northern and southern populations be recognized as subspecies (Figs 2–3). Thus, Penrith (1984) designated southern populations as the nominate subspecies and named the northern populations *Onymacris unguicularis schulzeae*—honoring Lieselotte Prozesky-Schulze, who first reported differences between northern/southern populations on the basis of larval characteristics (Schulze 1964). Penrith’s (1984) view of subspecies reflects the classic use of this taxonomic category in recognizing “geographic forms which cannot rank as full species” by noting that her morphological diagnosis could not “on the present evidence, separate the northern population more than subspecifically from southern populations.”

Efforts in subspecies delimitation mirror those of species delimitation conceptually if not methodologically and, similarly, engage controversy (Mayr and Ashlock 1991; Mortiz 1994; Burbrink et al. 2000; Zink 2004; Cronin 2006; Phillimore and Owens 2006; Jorgensen et al. 2013). Despite contention over subspecific rank, its taxonomic utility, and evolutionary validity, the category nonetheless remains the sole infraspecific unit recognized by the International Code of Zoological Nomenclature (ICZN 1999). Moreover, certain animal groups (e.g., birds, butterflies, beetles) still contain significant numbers of traditionally-recognized subspecies. Braby et al. (2012) recently provided a critical update of the subspecies concept, justifying viability and recommending that it correspond closely in theory and practice to the general lineage species concept (de Queiroz 1998, 2007). They proposed its application be restricted to extant groups of populations “representing partially isolated lineages of a species that are allopatric, phenotypically distinct, have at least one fixed diagnosable character state, and that these character differences are correlated with evolutionary independence according to population genetic structure.”

Subspecific taxa are now routinely reassessed using molecular phylogenetic analysis as a key component of integrative or coalescent approaches to recover evolutionarily independent lineages. These investigations generally yield one of two outcomes. Patterns of genetic variation may exhibit discordance with traditionally defined subspecies, either phenotypically or geographically, if not both (Burbrink et al. 2000, Zink 2004, Joyce et al. 2009, Spinks et al. 2013). In effect, the subspecies fail to be recovered as historically independent lineages—their morphological distinctions being reinterpreted as local adaptation, clinal variation, etc.—and are dismissed as valid taxonomic entities. Alternatively, genetic differentiation corroborates the phenotypic variation defining subspecies, in which case researchers may justify trinomial retention (Braby et al. 2012), elevation to full species (Glor and Laport 2012), or some combination thereof (Fuchs et al. 2011).

In this study we examined morphological and genetic variation in *Onymacris unguicularis*, adopting Braby et al.’s (2012) criteria to evaluate the validity of its polytypic status. Our inquiry involved a reassessment of Penrith’s (1984) diagnostic morphological characters in conjunction with (1) morphometric analysis of additional phenotypic variation and (2) phylogenetic analysis of mitochondrial DNA sequences.

## Materials and methods

### Morphological analysis

**Figures 4–7.**
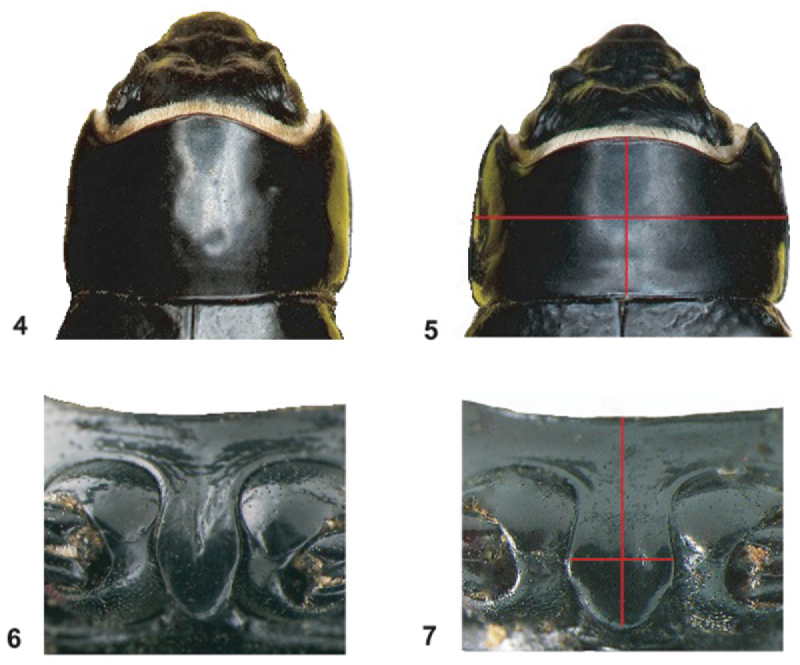
Pronotum (**4–5**) and prosternum (**6–7**) of *Onymacris unguicularis unguicularis* (**4**, **6**) and *Onymacris unguicularis schulzeae* (**5**, **7**). Measurements for ratio calculations are marked on **5** and **7**.

**Figures 8–9.**
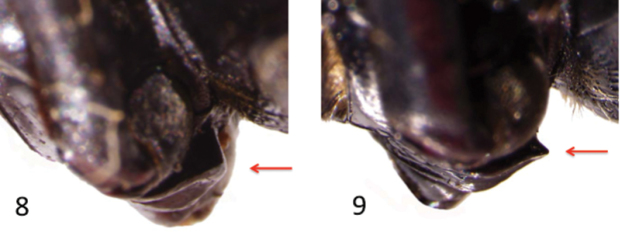
Lateral aspects of the prosternal process depicting a blunt apex (**8**) in *Onymacris unguicularis schulzeae* and toothed apex (**9**) in *Onymacris unguicularis unguicularis*.

Penrith’s (1984) morphological evidence for subspecific recognition involved shape differences of the pronotum and prosternal process (Figs 4–7). The pronotum is more strongly transverse in *Onymacris unguicularis schulzeae*, and its prosternal process is generally broader, featuring a blunt apex that is largely hidden in lateral aspect (Fig. 8). Conversely, the prosternal process in *Onymacris unguicularis unguicularis* is evident in lateral aspect, its apex often appearing as tooth-like projection (Fig. 9).

To reassess Penrith’s (1984) diagnostic morphological characters and explore additional aspects of phenotypic variation, we examined a series of pinned specimens from the Ditsong National Museum of Natural History (formerly Transvaal Museum), Pretoria, South Africa. Material included 93 *Onymacris unguicularis unguicularis* representing 11 populations and 30 *Onymacris unguicularis schulzeae* (all paratypes) representing four populations (Appendix). Two and five additional specimens of *Onymacris unguicularis unguicularis* and *Onymacris unguicularis schulzeae*, respectively (representing a portion of our genetic sample), were also examined. Specimens were photographed to provide dorsal and ventral images of each beetle. Images were made using a Visionary Digital Imaging System (Visionary Digital^TM^, Richmond, VA).

Penrith (1984) quantified pronotal shape differences between subspecies as a ratio of pronotal length divided by pronotal width (PL/PW). We repeated these measurements as part of our morphological analysis, and also quantified differences in the prosternal process as a length/width ratio (Figs 5 and 7), using the software program Image J. Additionally, Penrith (1984) noted possible differences in elytral shape, which appears to be “less elongate, broader, and more abruptly tapered posteriorly” in *Onymacris unguicularis schulzeae*. We therefore used a geometric morphometric analysis to assess putative differences in dorsal (elytral) shape. Mindful that sexual dimorphism may contribute to elytral shape variation, we acknowledge its potential to confound signal attributable to subspecific variation. However, only one species of *Onymacris*, *Onymacris plana* (Péringuey, 1888), exhibits pronounced sexual dimorphism in elytral shape; in all others the female’s elytra are only “slightly broader than those of the male,” with “much overlap” (Penrith 1975). In *Onymacris unguicularis*, frequency distributions of maximal elytral width, expressed as a percentage of elytral length, exhibit complete overlap between the sexes (Penrith 1975), and elytral shape in northern populations has been dismissed as being “scarcely dimorphic” (Penrith 1984). We should note that sexual dimorphism is evident in *Onymacris unguicularis*: males possess longer legs and, uniquely within *Onymacris*, bear setose brushes on the anterior femora (Penrith 1975). Given the species’ limited elytral dimorphism and our relatively small sample of *Onymacris unguicularis schulzeae* (n = 35), we elected to combine the sexes in our morphometric analysis.

**Figure 10.**
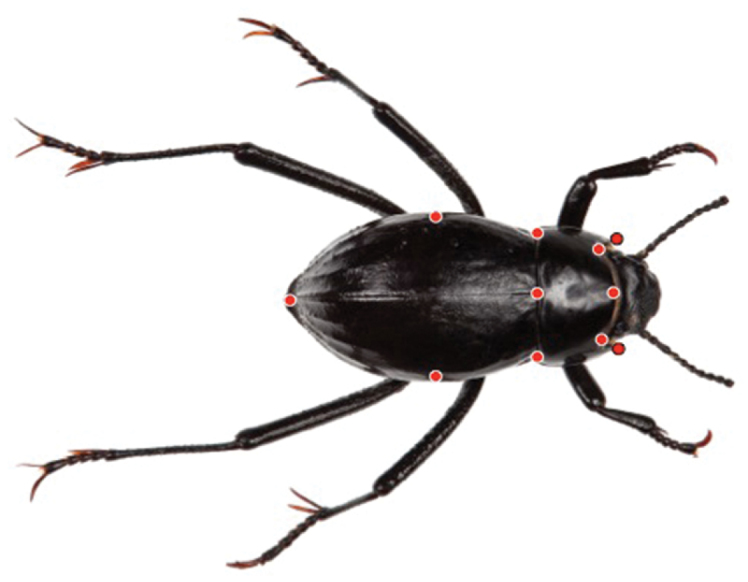
Landmarks for the geometric morphometric analysis of dorsal (elytral) shape.

We identified eleven dorsal landmarks—eight type 1 and three type 3 (Brookstein 1991; Fig. 10)—and used the programs tps-Util and tps-DIG2 (Rohlf 2012) to assemble dorsal (elytral) image files for analysis and score landmarks, respectively. Landmarks were aligned and scaled to size using the generalized least squares Procrustes superimposition method (Rohlf and Slice 1990), which removes information not relevant to shape (location, scale, and rotational effects). Relative warps were calculated with *α* set to zero, thus weighting all principal warps equally. Superimposition, calculation of relative warps, and calculation of centroid size were preformed using the program MorphoJ (Klingenberg 2011).

### Molecular phylogenetic analysis

Sixteen beetles were captured, preserved (100% ethanol), and processed for DNA analysis; the specimens included 12 *Onymacris unguicularis unguicularis*, representing three geographic localities, and four *Onymacris unguicularis schulzeae*, representing two relatively close localities (Fig. 1; Appendix). *Onymacris laeviceps* Gebien, 1938 and *Onymacris plana*, identified as sister taxa to *Onymacris unguicularis* in a generic-level phylogeny (Lamb and Bond 2013), served as outgroups. The mitochondrial genes cytochrome oxidase I (*cox1*) and cytochrome oxidase II (*cox2*) were amplified using the primers and PCR conditions listed in Table 1.

**Table 1.** PCR primers and amplification conditions.

Gene	Primer	Annealing	Cycles	Reference
*cox1*	TY-J-1460	50oC	35	Simon et al. (1994)
	TL2-N-3014			
	C1-J-2183	sequencing only		
*cox2*	TL2-J-3037	50oC	35	
	TK-N-3785			

Amplification products were cleaned using exoSAP-IT (USB Corp.) and sequenced on an Applied Biosystems 3130 capillary sequencer. Sequences were edited and assembled in Sequencher 4.9 (GeneCodes, Ann Arbor, MI) and aligned using ClustalX ver. 2.0 (Larkin et al. 2007), after which sequences were translated to ensure a correct reading frame.

We used Bayesian inference (BI) and maximum likelihood (ML) methods to analyze the concatenated gene (*cox1-cox2*) dataset. We used Kakusan 4 (Tanabe 2007) to select nucleotide substitution models for BI, partitioning protein-coding genes by codon position and assessing each gene/codon partition using the Bayesian Information Criteria (BIC4 criterion). BI analysis was conducted in MrBayes 3.1.2 (Ronquist and Huelsenbeck 2003) and involved two concurrent runs of four simultaneous Markov Chain Monte Carlo chains for 20,000,000 generations, with trees sampled every 1,000 generations. Topologies in the first 25% of the posterior distribution were discarded as burn-in. Likelihood values for all post-analysis trees and parameters were evaluated for convergence and burn-in using the “sump” command in MrBayes and the computer program Tracer ver. 1.5 (Rambaut and Drummond; http://evolve.zoo.ox.ac.uk/software.html?id=tracer). Trees remaining after burn-in were used to calculate posterior probabilities using the “sumt” command. The ML analysis, executed in RAxML ver. 7.2.8 (Stamatakis 2006), comprised 1,000 random sequence addition replicates (RAS) using the commands “-# 1000” and “–m GTRGAMMA.” Bootstrap support values were calculated using the same search parameters with 1,000 replicates, and bootstrap results were applied to the best tree recovered in the RAS search.

## Results and discussion

### Morphometrics

**Figure 11.**
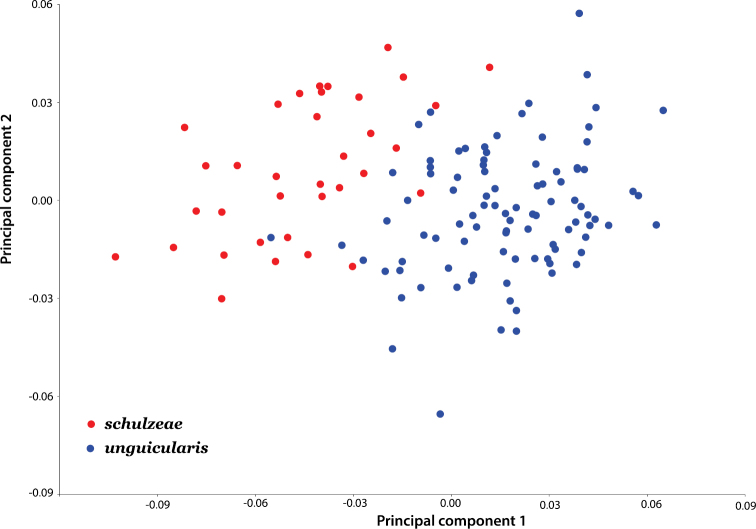
Scatterplot of the principal component scores derived from geometric morphometric analysis of dorsal shape.

Ratios generated for both the protonal and prosternal datasets differed significantly between subspecies (p < 0.0001), with minimal overlap for each character (Table 2). In the geometric morphometric analysis, the first two principal components based on the non-uniform components of dorsal (elytral) shape account for 78.54% of the variation between subspecies. An ordination plot of PC1 and PC2 revealed that the two subspecies are relatively well separated along the PC1 axis (Fig. 11). Dorsal shape separation probably reflects proportionally longer elytra in *Onymacris unguicularis unguicularis*, which become apparent in side-by-side comparisons with *Onymacris unguicularis schulzeae* (Figs 2–3). In light of these findings, we measured elytral length and width (at the midpoint of elytral length) for all specimens to determine whether a simple ratio (EL/EW) might reflect the subspecific separation observed in our geometric morphometric analysis. We also noted the position of greatest elytral width for each specimen, scored as midpoint, anterior to midpoint, or posterior to midpoint. Despite broad overlap, elytral ratios differed significantly (p < 0.0001) between subspecies (Table 2); elytral width is widest anterior to midpoint in both subspecies but is positioned closer to the pronotal suture in *Onymacris unguicularis schulzeae*. Of the three ratios, we consider that for the pronotum to be the strongest diagnostic metric.

**Table 2.** Pronotal, prosternal, and elytral ratio means and ranges.

Character	Subspecies	N	Mean	Range
pronotum	*Onymacris unguicularis unguicularis*	95	1.66 ± 0.08	1.47–1.83
	*Onymacris unguicularis schulzeae*	35	1.97 ± 0.13	1.73–2.35
prosternum	*Onymacris unguicularis unguicularis*	94	2.22 ± 0.17	1.86–2.71
	*Onymacris unguicularis schulzeae*	33	2.01 ± 0.14	1.65–2.34
elytra	*Onymacris unguicularis unguicularis*	95	1.44 ± 0.08	1.25–1.61
	*Onymacris unguicularis schulzeae*	34	1.35 ± 0.07	1.24–1.47

### Molecular phylogenetics

**Figure 12.**
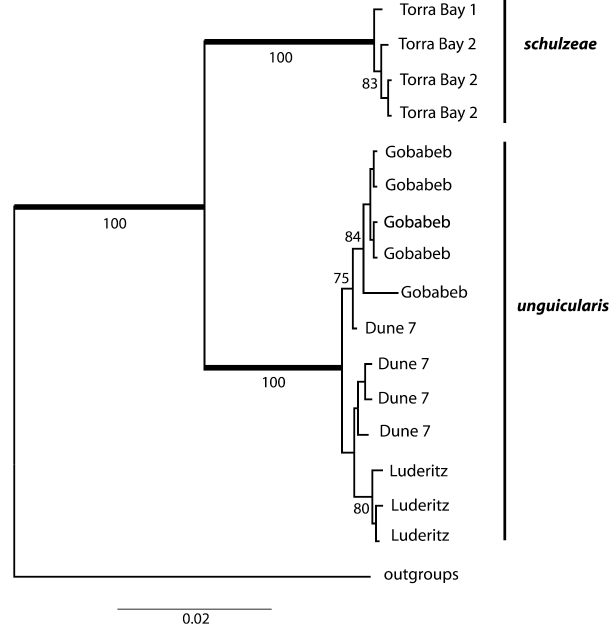
Bayesian consensus tree showing relationships within *Onymacris unguicularis*. Bold branches subtend nodes with Bayesian posterior probabilities of 1.0; numbers below the branches are ML bootstrap values.

Concatenated sequence data for the *cox1* (1574 bp) and *cox2* (680 bp) genes yielded eleven haplotypes among the 16 beetles surveyed: eight haplotypes for *Onymacris unguicularis unguicularis* and three for *Onymacris unguicularis schulzeae* (Genbank accession numbers KF835703-KF835721). No haplotypes were shared between subspecies. Mean haplotype divergence (calculated from uncorrected pair-wise distance values) was limited across geographic localities for both *Onymacris unguicularis unguicularis* (*cox1* = 0.046%; *cox2* = 0.027%) and *Onymacris unguicularis schulzeae* (*cox1=* 0.008%; *cox2* = 0.014%) but differed substantially between the two subspecies (*cox1 =* 3.87%; *cox2* = 3.01%). The BI (harmonic mean –ln = 4690.15) and ML (–ln = 4172.41) analyses generated topologically identical trees in which subspecies were shown to be reciprocally monophyletic (Fig. 12). Moreover, subspecific monophyly was strongly supported (Bayesian posterior probabilities = 1.0; ML bootstraps = 100%), in contrast to the marginal to moderate support observed for haplotype relationships of geographic localities within subspecies (Fig. 12).

### Conclusions

We employed Braby et al.’s (2012) integrative approach to evaluate the polytypic status of *Onymacris unguicularis* and found support for each criterion in their template for subspecies delimitation. *Onymacris unguicularis* is an ultrapsammophile confined to major dune fields within the northern (Cunene, Skeleton Coast) and southern (Namib) sand seas. Separated by 300 km of unsuitable substrate, these populations are unquestionably allopatric, satisfying Braby et al.’s first criterion. Regarding criterion two, phenotypic distinctiveness, we confirmed qualitative differences in pronotal and prosternal shape and verified putative distinctions in elytral shape. Patterns in larval variation—the ninth abdominal tergum being shorter and broader in northern populations (Schulze 1964)—complement differences in adult morphology and augment the case for phenotypic distinctiveness. Support for Braby et al.’s third criterion, character difference correlations with genetic variation, involves a phylogeographic profile that is perfectly congruent with the north-south partition in phenotypic variation. More importantly, the reciprocal monophyly observed between northern and southern haplotypes (with associated levels of genetic divergence) identifies respective paths of evolutionary independence. These data demonstrate that northern and southern populations of *Onymacris unguicularis* are phylogenetically distinct under the general lineage concept. Thus, we endorse Penrith’s (1984) taxonomic interpretation in recognizing *Onymacris unguicularis unguicularis* and *Onymacris unguicularis schulzeae* as valid taxa.

## Appendix

**Table:** Localities for genetic and morphometric samples.

Subspecies	Dataset	N	Locality	Voucher numbers*
*Onymacris unguicularis unguicularis*	morphometric	10	Anigab	
		9	Blauberg	
		10	Bogenfels	
		1	Chaneis	
		12	Gobabeb	
		8	Grillental	
		9	Hottentot Bay	
		5	Luderitz	
		10	Spencer Bay	
		10	Swakopmund	
		11	Walvis Bay	
	genetic	4	Dune 7, near Walvis Bay; -22.9691, 14.5946	TL022 TL023 TL024 TL025
		5	Gobabeb; -23.5691, 15.0424	TL026 TL027 TL028 TL029 TL030
		3	20 km E Luderitz; -26.7110, 15.2828	TL031 TL032 TL033
*Onymacris unguicularis schulzeae*	morphometric	3	near Foz du Cunene, Angola	
		4	Lacrau, 13 km N Fos du Cunene, Angola	
		11	Kaokoveld coast, between Koichab and Unjab rivers	
		12	Unjab River, 8 km from mouth	
	genetic	5	near Torra Bay; -20.3345, 13.2929	
		3	near Torra Bay; -20.3345, 13.2929	TL034 TL035 TL036
		1	near Torra Bay; -20.2738; 13.2655	TL037

*as coded in Genbank
